# Mechanisms of CaMKII Activation in the Heart

**DOI:** 10.3389/fphar.2014.00059

**Published:** 2014-04-02

**Authors:** Jeffrey R. Erickson

**Affiliations:** Department of Physiology, University of OtagoDunedin, New Zealand

**Keywords:** CaMKII, heart failure, oxidative stress, diabetes, *O*-GlcNAc modification

## Abstract

Calcium/calmodulin (Ca^2+^/CaM) dependent protein kinase II (CaMKII) has emerged as a key nodal protein in the regulation of cardiac physiology and pathology. Due to the particularly elegant relationship between the structure and function of the kinase, CaMKII is able to translate a diverse set of signaling events into downstream physiological effects. While CaMKII is typically autoinhibited at basal conditions, prolonged rapid Ca^2+^ cycling can activate the kinase and allow post-translational modifications that depend critically on the biochemical environment of the heart. These modifications result in sustained, autonomous CaMKII activation and have been associated with pathological cardiac signaling. Indeed, improved understanding of CaMKII activation mechanisms could potentially lead to new clinical therapies for the treatment or prevention of cardiovascular disease. Here we review the known mechanisms of CaMKII activation and discuss some of the pathological signaling pathways in which they play a role.

## CaMKII STRUCTURE/FUNCTION RELATIONSHIP

Calcium/calmodulin (Ca^2+^/CaM) dependent protein kinase II (CaMKII) is expressed as a multimeric protein, typically comprised of 12 subunits in most commonly observed physiological conditions (Hoelz et al., 2003). Individual monomers assemble into a dodecameric mulitmer via association at the *C*-terminal domain of each subunit. This association takes the form of a pair of hexameric rings arranged in parallel with one another (Rellos et al., 2010). This CaMKII structural motif is sometimes termed a “wagon wheel” arrangement, as individual monomers form the spokes of the wheel around a central core.

**FIGURE 1:**
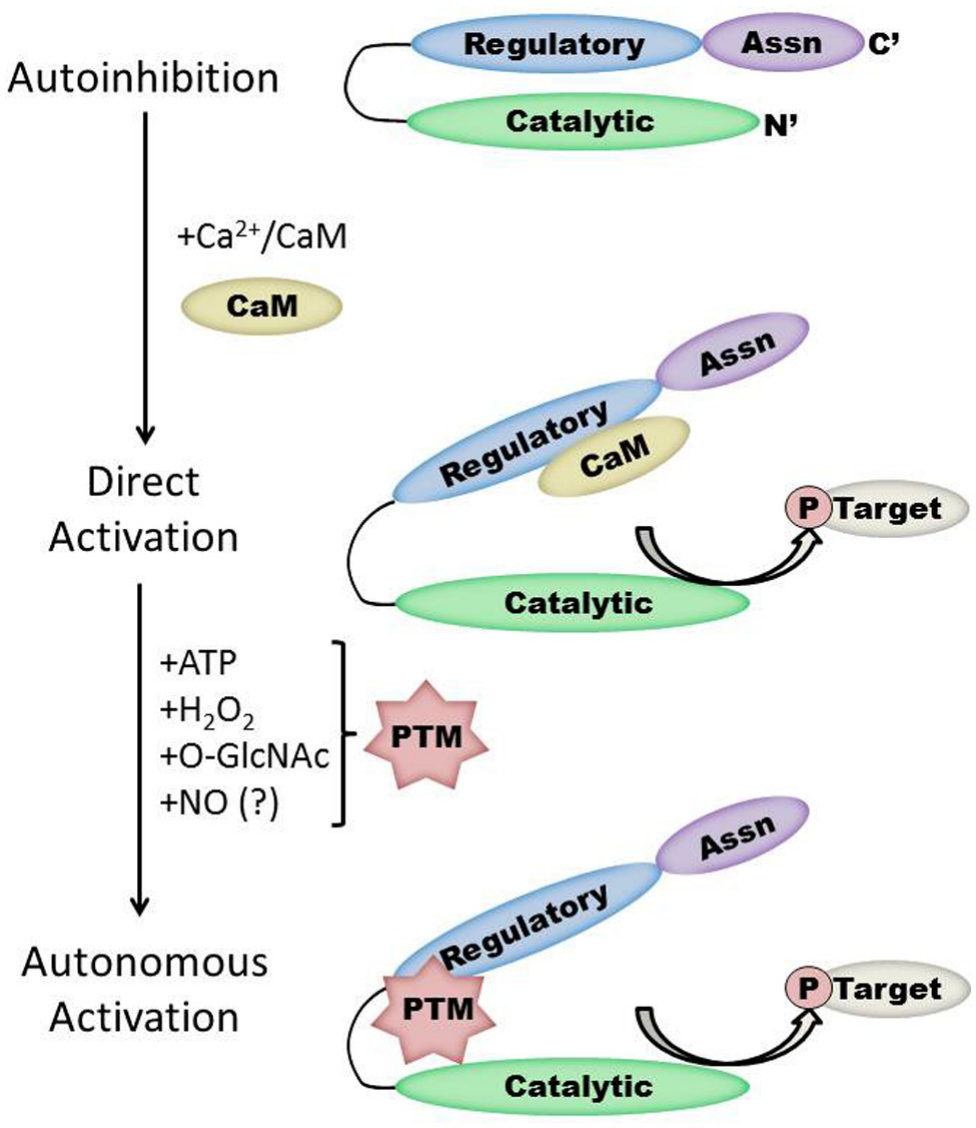
**Schematic representation of the CaMKII structure/function relationship in the heart.** Under basal conditions, the kinase is autoinhibited due to interaction between the regulatory and catalytic domains. Direct activation occurs when Ca^2+^/CaM binds to CaMKII, while subsequent post-translational modification (PTM) results in autonomous kinase activity. Protein interactions with CaMKII autonomously activate CaMKII in the brain but have not yet been demonstrated in the heart. Note that the size of each region is not to scale and will vary by isoform and splice variant of CaMKII.

**FIGURE 2:**
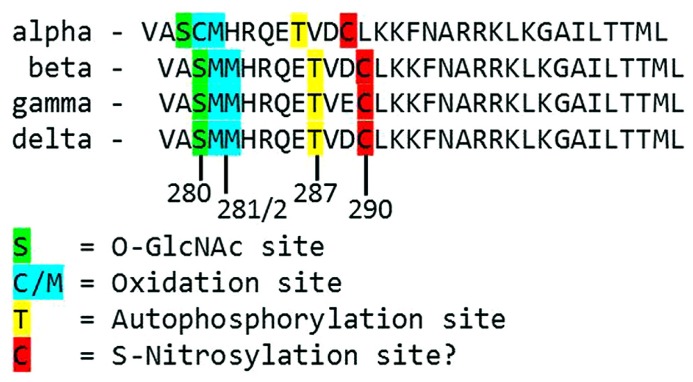
**Alignment of the primary structure of regulatory domains from *Mus musculus* CaMKII isoforms.** Specific residues associated with autonomous activation by post-translational modifications have been labeled.

Each monomer is itself comprised of three domains (**Figure 1**). The *C*-terminal association domain directs assembly of the CaMKII multimer, while the *N*-terminal catalytic domain binds to potential substrates and performs the serine/threonine kinase function of CaMKII. In the intervening linker region (the composition of which varies greatly depending on isoform and splice variant) lies the regulatory domain, which has two primary roles. First, the regulatory domain acts as a substrate for the catalytic domain within each CaMKII monomer, while adjacent regulatory domains within the multimer block both substrate and ATP binding to the catalytic domain itself (Rosenberg et al., 2005). Thus, the regulatory and catalytic domains are tightly associated at basal conditions, resulting in autoinhibition of the kinase. Secondly, the regulatory domain binds CaM with a K_ D_ of 10–70 nM (Gaertner et al., 2004) when intracellular [Ca^2+^] is elevated (half maximal CaM occupancy requires approximately 1.0 μM Ca^2+^; Rostas and Dunkley, 1992). When CaM binds to CaMKII, a conformational shift occurs that disrupts the association between the catalytic and regulatory domains, exposing the catalytic domain for substrate binding and relieving autoinhibition of the kinase. CaMKII function is critically linked to this CaM binding function of the regulatory domain; indeed, all known mechanisms of CaMKII activation require Ca^2+^/CaM binding as an initiating step. Thus, the extent of CaMKII activation within a cell is likely to be at least partly dependent on local CaM concentrations, as has been proposed in cardiac myocytes (Saucerman and Bers, 2008). The role of the regulatory domain to CaMKII function is so integral, it is not surprising to discover that the known isoforms and splice variants of mammalian CaMKII have nearly identical primary structure within the regulatory domain (**Figure 2**).

## CaMKII PHOSPHORYLATION AND AUTONOMOUS ACTIVITY

The duration of CaMKII activation by Ca^2+^/CaM binding is dependent on the frequency of Ca^2+^ release events (De Koninck and Schulman, 1998). Conditions of prolonged Ca^2+^/CaM association with CaMKII, as would be observed during lengthy or high frequency calcium transients, allow for intersubunit autophosphorylation of CaMKII monomers at the T287 (T286 in the α isoform) site. The addition of a phosphate group at T287 has two critical effects on CaMKII function. First, the binding of affinity of CaM for the CaMKII regulatory domain increases by more than 1000-fold (Meyer et al., 1992). Second, the presence of a negatively charged phosphate group at the T287 site precludes reassociation of the catalytic and regulatory domains, preventing autoinhibion even if [Ca^2+^]_ i_ falls and CaM dissociates from CaMKII (Lai et al., 1987). Subsequently, autonomous activation of CaMKII by T287 phosphorylation will persist until the phosphate group is removed by a protein phosphatase (PP1 or PP2A; Stack et al., 1997). The extent of CaMKII activation in the heart after autophosphorylation is not known, though evidence in the neuronal form of CaMKII points to autonomous activity that corresponded to less than 25% of full CaMKII activity (Coultrap et al., 2010).

CaMKII has been called the “memory molecule” in part for its function in the processing of neural information from short-term to long-term memory, but also due to the ability of CaMKII to translate Ca^2+^ transient frequency into autonomous activation of the kinase (Hudmon and Schulman, 2002; Coultrap and Bayer, 2012). Indeed, the discovery that post-translational modification of CaMKII resulted in a shift from Ca^2+^-dependent to Ca^2+^-independent states gave rise to the now popular belief that CaMKII functions as a nodal cardiac signaling molecule that translates upstream cellular events into downstream physiological effects. In the heart, autophosphorylation of CaMKII is particularly prevalent during β-adrenergic signaling (Erickson et al., 2011) and is thought to play a key role in the development of cardiac hypertrophy and dilated cardiomyopathy (Zhang et al., 2003).

## CaMKII OXIDATION

While direct Ca^2+^/CaM-binding and T287 autophosphorylation have been recognized for over two decades, more recently published results have demonstrated new pathways of CaMKII activation. For example, CaMKII can be directly modified by reactive oxygen species (ROS), resulting in autonomous activation (Erickson et al., 2008). The regulatory domain of CaMKII contains a pair of redox-sensitive amino acids (C280/M281 in the α isoform, M281/M282 in the β, γ, and δ isoforms) that can be oxidized when exposed to elevated levels of oxidative stress. As is the case in T287 phosphorylation, redox modification of CaMKII only occurs after Ca^2+^/CaM binding initiates a conformational shift of the inhibited kinase and exposes the target sites. An examination of the crystal structure of CaMKII suggests that the addition of an oxidative modification at the M282 site preserves kinase activity by preventing reassociation between the regulatory and catalytic domains (Rellos et al., 2010). Additionally, this study suggests that M282 oxidation should preclude T287 phosphorylation, though this hypothesis has not been confirmed experimentally. Mutation of the M281 and M282 residues does not disrupt phosphorylation-dependent CaMKII activation (Erickson et al., 2008). We can therefore conclude that while the phosphorylation- and oxidation-dependent mechanisms share similarities, they constitute parallel pathways for maintaining CaMKII activation.

Since the redox-dependent mechanism was reported, oxidation of CaMKII has been found to play a role in a number of cardiac pathologies. For example, the renin-angiotensin-aldosterone signaling (RAAS) pathway promotes enhanced oxidative stress in the heart. Angiotensin II (AngII) induced apoptosis of cardiac myocytes is ablated in isolated neonatal mouse myocytes expressing the oxidant-resistant mutant of CaMKII (Erickson et al., 2008). Similarly, elevated levels of circulating aldosterone enhance CaMKII oxidation, leading to apoptosis, impaired cardiac function, and potential cardiac rupture (He et al., 2011; Velez Rueda et al., 2012). Redox-dependent CaMKII activation also enhances electrical remodeling in the heart, including enhanced Na^+^ current (and subsequent [Na^+^]_ i_ overload; Wagner et al., 2011), apoptosis of sinoatrial nodal cells (Swaminathan et al., 2011), and eventual impaired conduction of transients (Christensen et al., 2009), all of which contribute to life-threatening arrhythmias. Moreover, a diabetic mouse model expressing oxidation resistant CaMKII (MM281/282VV) was found to be resistant to sinoatrial nodal cell death, fibrosis, and mortality after myocardial infarction compared to diabetic animals expressing wild type CaMKII (Luo et al., 2013). These observations suggest that oxidation-dependent CaMKII activity plays a critical role in numerous pathological processes in the heart.

Just as T287 phosphorylation of CaMKII is reversed by phosphatases, the enzyme methionine sulfoxide reductase A (MsrA) reduces oxidized methionine residues to inactivate the kinase (Erickson et al., 2008). Thus, MsrA has emerged as a potential cardioprotective molecule against the deleterious effects of oxidative stress. For example, a genetic knockout mouse model lacking MsrA was found to have significantly increased redox-activated CaMKII, myocyte apoptosis, structural remodeling, and mortality 4 weeks after myocardial infarction compared to wild type littermates (Erickson et al., 2008). Conversely, a transgenic mouse model that overexpresses MsrA was found to be less susceptible to aldosterone-mediated CaMKII oxidation and cardiac remodeling (He et al., 2011), and was also more resistant to AngII-induced atrial fibrillation (Purohit et al., 2013). Interestingly, the expression and activity of MsrA is reduced in brain tissue from aging human patients (Petropoulos et al., 2001), and this age-dependent reduction in MsrA activity is linked with the development of Alzheimer’s disease (Gabbita et al., 1999). However, the age-dependence of MsrA expression and activity in the human heart has not been studied.

## CaMKII ACTIVATION IN DIABETES

Diabetes mellitus is marked by a number of altered cellular signaling pathways, creating physiological stress that can activate CaMKII. For example, diabetic patients have a significantly greater proportion of oxidized to total CaMKII compared to non-diabetic patients (Luo et al., 2013), consistent with observations that altered cellular metabolism in diabetics results in enhanced oxidative stress. A recently published study points to a novel mechanism for CaMKII activation during hyperglycemia and diabetes through the addition of an *O*-linked *N*-acetylglucosamine (*O*-GlcNAc) modification (Erickson et al., 2013). Post-translational modification by *O*-GlcNAc (“*O*-GlcNAcylation”) is an emerging field with important regulatory implications in disease states characterized by altered glucose signaling, such as myocardial infarction and diabetes (Hart et al., 2007; Chatham and Marchase, 2010). *O*-GlcNAc modification can alter protein function (Dias et al., 2009), and such regulation is known to play a role in both the heart and brain (Gao et al., 2001). *O*-GlcNAcylation is catalyzed by the enzyme *O*-GlcNAc transferase in the presence of the substrate UDP-*N*-acetylglucosamine, which is produced in conditions of elevated glucose as a product of the hexosamine biosynthesis pathway (Hart et al., 2007).

When CaMKII is activated by Ca^2+^/CaM binding in the presence of elevated [glucose], an *O*-GlcNAc modification is added to the regulatory domain at S280 (S279 in the alpha form), resulting in autonomous activation of the kinase (Erickson et al., 2013). The extent of both *O*-GlcNAc modification and activation of CaMKII varies with glucose availability in a dose-dependent manner and is reversible by the action of *O*-GlcNAcase, an enzyme that removes *O*-GlcNAc modifications from proteins. These observations point to a potential regulatory role for *O*-GlcNAcylation of CaMKII, consistent with the observation that the ratio of *O*-GlcNAc modified CaMKII to total CaMKII is greatly enhanced in the heart and brain from diabetic patients. Further, glucose induced Ca^2+^ leak from the sarcoplasmic reticulum (measured as Ca^2+^ sparks) is both CaMKII and *O*-GlcNAc dependent, suggesting a connection between *O*-GlcNAc mediated CaMKII activity and arrhythmogenesis in the diabetic heart. Indeed, pharmacological inhibition of the hexosamine biosynthesis pathway (and therefore the production of *O*-GlcNAc precursors) prevented ventricular tachycardia in hearts from diabetic rats challenged with dobutamine and caffeine (Erickson et al., 2013). Taken together, these observations suggest that *O*-GlcNAc modification of CaMKII could play a critical role in structural and electrical remodeling in the diabetic heart.

## ALTERNATE MECHANISMS OF CaMKII ACTIVATION IN THE HEART

The preceding sections describe several established mechanisms of CaMKII activation in the heart, but it is unlikely that they are exhaustive. Indeed, emerging evidence points to other potential mechanisms that are not fully described for cardiac CaMKII. For example, several recent studies have provided new evidence suggesting the existence of an undescribed mechanism of CaMKII activation mediated by intracellular nitric oxide (NO). For example, isoproterenol-induced Ca^2+^ leak from the sarcoplasmic reticulum is determined in part by the activity of both NO synthase and CaMKII (Curran et al., 2009). ATP-sensitive potassium channels are also modulated by NO, and these NO-dependent effects on K_ ATP_ were ablated in knockout mice lacking the cardiac isoform of CaMKII (Zhang et al., 2013). CaMKII activity is enhanced in the presence of the NO donors NOC-18 (Zhang et al., 2013) and GSNO [Gutierrez et al., 2013; though another study found inactivation of CaMKII by NO donors (Song et al., 2008)], while a non-site specific antibody suggests that, at least *in vitro*, CaMKII contains S-nitrosylated cysteine residues. Computational prediction of S-nitrosylation sites on CaMKII indicate three potential target sites, including the C290 site present in the regulatory domain (Gutierrez et al., 2013). While none of the potential sites have been confirmed and the mechanism by which nitrosylation induces CaMKII activity has not been described, the evidence in favor of this novel pathway of CaMKII activation is compelling.

Additional potential mechanism for activation of CaMKII in the heart is through interaction of the kinase with protein partners. The first and best described such mechanism was identified in the α (neuronal) isoform of CaMKII, which can become autonomously activated through association with the NMDA receptor (Strack and Colbran, 1998; Bayer et al., 2001), contributing to long term potentiation (Barria and Malinow, 2005), and memory consolidation (Halt et al., 2012). While this topic extends beyond the scope of the current, cardiac-focused review [please see references Coultrap and Bayer (2012) and Colbran (2004) for a closer examination of CaMKII/NMDA receptor binding], it does suggest the possibility that interactions between CaMKII and other protein partners could alter kinase activity. For example, binding of α-actinin to the α isoform of CaMKII can mimic Ca^2+^/CaM binding and activate the kinase in HEK293 cells (Jalan-Sakrikar et al., 2012). In cardiac cells, CaMKII association with the K_ V_4.3 potassium channel blocks Ca^2+^/CaM binding and reduces kinase activity (Keskanokwong et al., 2011), but whether protein binding can enhance CaMKII activity in the heart is an important open question.

## SYNERGISTIC ACTIVATION OF CaMKII: AN OPEN QUESTION

While our understanding of the underlying mechanisms that determine CaMKII function has grown rapidly of late, numerous questions remain unanswered. Recent efforts have focused on the characterization of individual pathways of CaMKII activation and on the downstream pathological consequences of these specific pathways. In the context of cardiac disease states with complex pathophysiologies, such as in heart failure or diabetes, multiple signaling pathways are likely to be altered. Complicating matters is the issue that the various pharmacological inhibitors of CaMKII currently in use rely on different mechanisms. For example, the CaMKII inhibitor KN-93 (but not the inactive analog KN-92) competes with CaM binding to the CaMKII regulatory domain and prevents activation of the kinase (Hudmon and Schulman, 2002). However, KN-93 has no such CaMKII inhibitory effect after autonomous activation (Buard et al., 2010). Conversely, peptide inhibitors such as AIP and AC3-I, which mimic the regulatory domain sequence and bind to the catalytic domain of CaMKII, prevent substrate binding regardless of the mode of CaMKII activation. Thus, careful consideration must be given to the selection and development of inhibitors in studies focused on CaMKII (Coultrap and Bayer, 2011). For a more thorough review of CaMKII inhibitors, please see the review by Dr. Howard Schulman in this Research Topic.

Moreover, CaMKII itself contributes to the regulation of intracellular signaling processes such as mitochondrial function (Joiner et al., 2012) and insulin secretion (Dixit et al., 2013), many of which could form potential feedback loops through additional CaMKII modification. Thus, the interplay between various mechanisms of CaMKII activation, particularly during the development of cardiovascular diseases, is an important open line of inquiry. Moreover, models based on data generated from examination of the crystal structure of CaMKII predict that intersubunit phosphorylation does not occur as a random, coincidence-based process, but rather occurs via cooperative modification of individual subunits (Chao et al., 2010). This observation suggests that the activity of each CaMKII monomer is mediated acutely by the post-translational modifications present on adjacent monomers.

Does the presence of one type of modification enhance the probability of other modifications occurring on the same or on neighboring subunits? In one study enhanced *O*-GlcNAc modification of CaMKII was associated with an increase in phosphorylation of CaMKII in isolated rat myocytes exposed to hyperglycemia (Erickson et al., 2013). In summary, these results provide compelling evidence for potential cross-talk between signaling mechanisms, but more work will be required to elucidate a fully integrative model of CaMKII activation.

## CONCLUSION

Calcium/calmodulin dependent protein kinase II activity is dependent on a number of intracellular signaling pathways, either independently or as part of an integrative system. Thus, CaMKII represents an important nodal point in the translation of a broad range of cellular stresses into physiological and pathological pathways in the heart and other organ systems. Consequently, CaMKII has emerged as a potential therapeutic target in the clinical treatment of cardiac and neurological diseases. If we hope to develop a new generation of therapies based on modulation of CaMKII, we must understand the mechanisms that regulate CaMKII activity.

## Conflict of Interest Statement

The author declares that the research was conducted in the absence of any commercial or financial relationships that could be construed as a potential conflict of interest.
